# A qualitative assessment of practitioner perspectives post-introduction of the first Continuous Professional Competence (CPC) guidelines for emergency medical technicians in Ireland

**DOI:** 10.1186/s12873-015-0037-2

**Published:** 2015-05-24

**Authors:** Shane Knox, Suzanne Dunne, Walter Cullen, Colum P Dunne

**Affiliations:** Centre for Interventions in Infection, Inflammation & Immunity (4i), Graduate Entry Medical School, University of Limerick, Limerick, Ireland; Health Services Executive, National Ambulance Service College, Dublin, Ireland

**Keywords:** Emergency medical technicians, Continuous professional development, CPD, Continuous professional competence, CPC

## Abstract

**Background:**

In November 2013, the Irish Regulator for emergency medical technicians (EMTs) introduced the first mandatory requirement for registrants to demonstrate evidence of continuous professional development (CPD)/continuous professional competence (CPC). This qualitative study assessed the experience of practitioners with CPC-related materials provided to them by the Regulator in addition to identifying perceived or encountered practical challenges and suggested improvements six months following introduction of the requirement.

**Methods:**

Five fora were utilised, comprising two distinct groupings: a group of student EMTs (n = 62) and four discrete groups of qualified EMTs (total n = 131) all of whom had commenced the newly-introduced CPC process. All 193 volunteers were members of the Civil Defence (an auxiliary/voluntary organisation) and represented a nationwide distribution of personnel. Responses were categorised as ‘perceived’ challenges to CPC, relating to student EMTs, and ‘experienced’ challenges to CPC, relating to qualified EMTs. Responses also included suggestions from both groups of EMTs on how to improve the current system and guidance material. Audio/visual recordings were made, transcribed and then analysed using NVivo (version 10). A coding framework was developed which identified unifying themes.

**Results:**

All participants agreed that CPC for pre-hospital practitioners was a welcomed initiative believing that CPC activities would help ensure that EMTs maintain or enhance their skills and be better enabled to provide quality care to the patients they might encounter. Two specific areas were identified by both groups as being challenging: 1) the practicalities of completing CPC and 2) the governance and administration of the CPC process. Challenging practicalities included: ability of voluntary EMTs to gain access to operational placements with paramedics and advanced paramedics; the ability to experience the number of patient contacts required and the definition of what constitutes a ‘patient contact’. With regard to the governance and administration of CPC, it was suggested that in order to enhance the process, the Regulator should provide: an outline of the CPC audit process; examples of cases studies and reflective practice; templates for portfolios; and should establish a central hub for CPC information.

**Conclusion:**

These groups of Irish EMTs appeared keen to participate in continuous professional competence activities. In addition, these EMTs identified areas that, in their opinion, required clarification by the Regulator related to the practicalities of CPC and the governance and administration of CPC. More information, dissemination of sample requirements and further effective engagement with the Regulator could be used to refine the current CPC requirements for EMTs.

## Background

In Ireland, there are three levels of registration for practitioners of pre-hospital care: emergency medical technician (EMT), paramedic and advanced paramedic. In November 2013, the Regulator for pre-hospital (ambulance) practitioners in Ireland, the Pre-Hospital Emergency Care Council (PHECC), introduced a framework of Continuous Professional Development (CPD)/Continuous Professional Competence (CPC) targeting EMTs for the first time in Ireland. In doing so the Regulator was acknowledging the fact that EMTs represent the largest cohort of pre-hospital practitioners in Ireland and that while their focus was initially placed on this grouping CPC requirements for the smaller but more advanced practitioners, paramedics and advanced paramedics, would be enacted in 2016. The Regulator adopted CPC as the preferred term used to describe ongoing education and development for pre-hospital practitioners. The guidance provided to practitioners, by the Regulator, regarding CPC requirements, resulted from surveys and fora conducted and published by our group [1, 2]. The issuing of this guidance booklet was to assist EMTs in determining what was required of them in order to maintain their status as registered practitioners.

As of April 2014, there were 2200 EMT practitioners registered in Ireland, a small number of whom were members of state services (police, military or coast guard) but the majority of whom were associated with the St. John Ambulance, Civil Defence, Order of Malta Ambulance Corps, Irish Red Cross voluntary organisations. In Ireland, in order to qualify as an EMT, candidates must complete a five-week didactic training programme with an additional one week hospital and ambulance observer placement. This study involved facilitated fora comprised of EMTs from one of these organisations, the Civil Defence. The objective was to identify the perceived and/or actual challenges to compliance with CPC requirements experienced by those students or qualified EMTs, respectively, with a view towards such feedback informing the next iteration of Irish EMT CPC guidance.

There is evidence of CPD programmes for pre-hospital practitioners internationally which include the review and maintenance of competence. For example, in a study from Northern Norway, ambulance services described changes in terms of tasks performed and improving levels of competence [3] and, in Australia, the Council of Ambulance Authorities completed a project to develop paramedic professional competency standards [4]. In the United Kingdom, the Health Care Professions Council (HCPC), the Regulatory Body for healthcare professions, published guidelines for their many registered professions, including paramedics [5]. Martin [6] described the challenges of introducing CPD for paramedics in the UK which included: the need for paramedics to develop the intrinsic motivation required to assess their learning needs; their ability to respond accordingly; and the challenge for ambulance managers to facilitate opportunities for CPD. The Paramedic Association of Canada produced a national occupational competency profile for paramedics [7]. There is also evidence of consultation with registrants in other health care professions and determining of their opinions in relation to continuous professional development; *e.g.*, nurses [8–11], social workers [12], pharmacists [13] and radiographers [14, 15]. However, there is no evidence of such engagement with Irish pre-hospital practitioners on this subject except for our studies [2, 16, 17] and while there is evidence of guidelines on CPD internationally [18] and some evidence of engagement with healthcare professionals through professional liaison groups established by healthcare Regulators [19, 20] there is little evidence of specific engagement with pre-hospital practitioners prior to the introduction of CPD guidelines [21, 22].

This study aimed to provide information that would inform a revised, improved version of CPC guidelines for EMTs in Ireland in addition to being of interest to others internationally involved in designing or implementing CPD activities for healthcare professionals, especially pre-hospital practitioners.

## Methods

### Setting

In April/May 2014, approximately six months after the first introduction of CPC for EMTs practising in Ireland, one group of EMTs in training (n = 62) and four groups of qualified EMTs (total n = 131) were invited to participate in one of five facilitated fora.

The aim of this study was to identify perceived or encountered practical challenges and suggested improvements for the next iteration of CPC guidance for EMTs. Fora were utilised to allow feedback to predetermined open-questions. Fora, a form of group consultation with larger numbers than usually involved in focus groups, are designed to enable a facilitated discussion on specific topics, whereby participants were enabled to share and contribute to a wider discussion through a process of guided facilitation [23]. Such discussions have advantages for researchers in the field of health and medicine such that they encourage participation by people reluctant to be interviewed on their own or who feel they have nothing to say. As a result, fora and focus groups explicitly use group interaction as part of the method [24].

All participants had been asked, prior to the fora, to familiarise themselves with the CPC Guideline Booklet [1]. The design and conducting of the study were approved by the Ethics Committee of the Faculty of Education and Health Sciences, University of Limerick, Ireland and the Research Ethics Committee of the Health Services Executive Mid-Western Regional Hospital, Limerick, Ireland. All participants agreed to their inclusion in the research study by giving written consent.

The 62 EMT students constituted one group and convened on the first day. The other EMT groups (n = 131) convened over four separate days and consisted of: 20 participants on day one; 28 participants on day two; 40 participants on day three and 43 participants on day four.

The EMT student group comprised 40 females and 22 males with a large regional spread. The experienced group comprised 50 females and 81 males, also with a diverse regional spread. Broadly, there was nationwide representation with participation from 20 counties out of the national 26 counties over the five days.

There were a total of 273 registered EMTs within the Civil Defence at the time of convening the fora. Participants in this study represented 100% of the student EMTs within the Civil Defence, and 48% of the total registered EMTs within the organisation, at that time.

### Development of study instrument

The composition of the study instrument was based on a survey used with fora of pre-hospital practitioners prior to development of the original CPC guidelines [1, 2].

The study instrument: questions asked of participants:

1. Is CPC a good idea?
2. What problems do you foresee in completing CPC?
3. What is unclear about the related activities section in the CPC booklet for EMTs?
4. Learning Portfolio: what is your understanding of what a learning portfolio is?

### The fora

Each discussion forum lasted for approximately forty minutes and was facilitated by the Civil Defence College Principal (a trained facilitator), who is unconnected with this research. An introduction to the subject was provided by the facilitator and guidelines issued with regard to interaction and the objectives of the discussion forum. With the knowledge of the participants, the discussions were recorded on video. Questions were posed by the facilitator and reinforced using a multimedia presentation, whereby the question remained on the screen for all to see until that particular topic was concluded and agreement reached with the group to move on to the next question.

### Data analysis

All comments made by participants were transcribed from the recordings. These were not attributed to gender or to specific individuals. Using NVivo (version 10), participants’ responses from all the groups were initially coded to nodes defined by the four questions posed during the sessions (see Table 1 for coding framework). Two researchers completed the analysis (SD and SK) to allow for investigator verification of results. Transcripts were reviewed again to determine if any data were missed from the initial coding. Following this initial round of coding the data were further sub-classified within each of these codes into sub-codes of i) opinions, ii) questions, iii) suggestions and any other themes emerging. Further themes were identified, as stated in Table 1, which include ‘patient contact’, ‘duties’ and ‘practical challenges’.

**Table 1:** Selection of responses to the four questions posed to both cohorts: Student EMTs and Experienced EMTs (Nodes coded to are in bold)

**Question 1: Is CPC a Good Idea?**	
**Student EMTs**	**Experienced EMTs**
**Yes because:**	**Yes because:**
• “Because it keeps your mind ticking over you have to think about what you’re doing”	• “It keeps you up to date”
• “It keeps your skills up”	• “Helps you learn”
• “It is nearly a better thing for the voluntary organisations to do ‘cos we don’t get the same exposure”	• “Could be ready for any exam, it’s been 3 years since I’ve done my course”
	• “Keeps the skills up, keeps you on your toes, good to keep a record”
	• “If you are not getting patient contacts…you need to, to keep up to date
	• “It is absolutely necessary, it’s a good thing”
**Question:**	**Questions:**
“Is this model concrete or can it change?”	• “Do you see PHECC [Regulator]organising placements for us with HSE [Health Authority] so that we can get experience to build up our patient contact”
	**Suggestions:**
	• “I think it’s a good idea but as an EMT we are part of an organisation and we do work for the CDOs (Civil Defence Officers) but it would be a good thing to bring them up and tell them what we have to do, cos sometimes they’d just say ‘well that’s your problem, not ours’.”
	• “You could go to other counties to get events”
**Question 2: What problems to you foresee in completing CPC?**	
**Student EMTs**	**Experienced EMTs**
**Points for clarification:**	**Questions:**
• “What are we going to do if we go to events but don’t get any patient contacts?”	• “What are you looking recorded when you are recording patient contact; heart attack, what, what detail?”
• “If you go out and do loads of duties and no patient contact, if you keep a log … can that be counted?”	• “Is it from January to January”
• “Clarification required on mentoring, who does it, is it a non-instructor, can they mentor?”	• “Unsure what is expected from us”
• “There seems to be confusion with regard to when …we have to submit this. Every year or 18 months or every three years, this needs clarification”	• “What about stuff we did two years ago will PHECC [Regulator] count this?”
• “Who signs off on this? On our points?”	• “In relation to new EMTS are they still backdated from November 2013?
• “In relation to our training, we do a lot of first aid classes and we refresh all our training – that should be counted?”	• “One of them here says that you have to have mentoring…you get a point for each hour, but it must be signed by a paramedic or advanced paramedic…but what if you haven’t got one?”
• “What is the mechanism for audit?”	• **Duties:**
• “Should there be points for checking your ambulance equipment?	• “If you are working as a community first responder…can you count that?”
• “We are not in the ambulance service, all that we can get is minor …but no casualties contact, should that not count?”	• “If we put down a couple of duties we have done do we document it?”
• “Where do we get information regarding the seminars, conferences and activities where do we get this information? Should be on the PHECC [the Regulator] website like their calendar for exams”	• “Does this apply to our private lives or in our work then if we get a patient… like I work in the county council and if I get called as a first aider so should we be filling out PCRs (Patient Care Reports)?”
• “Continuous placements, ambulance placements, it’s all well and good training, training, training but unless you actually see real patients, you know”	• “Time on duty is different in the voluntary services”
• “Are you allowed to get CPC points from other organisations, I am also a member of the RNLI [The Royal National Lifeboat Institution], are we allowed to use their courses?”	**Patient contacts:**
• “How much do we need to do, is it 2 pages of a case study or ten pages, or how many words should it be?”	• “At least 12 patient contacts per year, what’s the situation if you can’t get the 12 patient contacts?”
• “How do we get a case study, we need patient contacts and a case study may be difficult for us?”	• “Is patient contact a completed PCR (patient care report) or ACR (ambulatory care report)?”
• “The evidence of at least 12 patient contacts per year, is that just like 12 plasters on 12 patients, what type of evidence do you need to show?”	• “If you’re in the situation where you are off duty and the ambulance service arrive, can you ask them for that PCR (patient care report) number as evidence of your patient contact?”
• “It is very important for EMTs to get ambulance placements. “	• “With regard to patient contact, I haven’t been out as I had a baby, so is that over the years, the 12 patient contacts or just for one year?”
• “Up-skilling [to new practice guidelines] if EMTs do refresher courses up-skilling will you get CPC recognition for that?”	• “Is the evidence of the patient contacts just the PCR (patient care report)? I’ve had the experience of the ambulance service were they don’t take mine but start a new one”
	• “If we are filling out pcrs, and the patients’ contact details are on it obviously should we be handing over the top copy to the paramedic, and we hold on to the second copy and you’re not in the Civil Defence you should cover your details…..?”
	• “From a duty point of view It’s hard to get 12 patient contacts, we are not out often enough in reality we don’t attend so many patients in a year”
	• “I’m out every weekend, but I won’t get 12 patients”
	• “We’re trying to keep the thing going at home, we are getting plenty of duties, we’re just not getting patients, so unless we start pushing people off horses or something…we’re not going to get patients”
	• “The 12 patient contacts per year, it’s too many”
	• “Like, we have 10 EMTs in the county like, that’s 120 patients a year, it just won’t happen”
	**Templates:**
	• “If there was a template that you just wanted to record… it would be good to complete rather than try to store Patient Care Reports”
**Practical challenges:**	**Practical Challenges:**
• “There is a need for more clarity in the booklet for an alternative to patient contact”	• “You should not be allowed to get 8 points for the one activity, it is not stated clearly that these 8 points cannot be all for one activity.”
• “With regard to Cardiac First Response [or CPR] recertifying, we only refresh every two years. Do we have to do more? This needs clarification”	• “Templates for case studies *etc.* its kinda vague”
**Suggestions:**	**Suggestions:**
• “It does not tell us where to go to get courses, it should tell us”	• “Examples of what should be in your portfolio, would be good”
• “More points for quality of care”	
• “What about scoring higher points if you have more serious casualties, as well as points for skills should there be, say, points for medications and stuff; cardiac chest pain compared to a plaster?”	**Comment:**
• “On the case study there should be key points or a template or for your reflective practice”	• “How do you know if I have made it up?”
	• “People working in the HSE [Health Authority] have the advantage of meeting more patients”
	• “I suppose it’s like the American system as well there is a difference between the volunteer EMTs and the professional EMTs (do you want a cert with Volunteer written on it?)What I’m saying is, we don’t spend our time out in ambulances, or in the back of ambulances we have other jobs, we don’t have the same chances…”
	• “Who will audit the whole process of CPC?”
**Question 3: What is unclear about the related activities section of the CPC booklet for EMTs (supplementary information is included in Table** **2** **)**	
**Student EMTs**	**Experienced EMTs**
**Coordination of CPC information:**	**Coordination of CPC information:**
• “How will we know about seminars and conferences and the costs of those?”	• “Where are the courses out there, how would you check that they are relevant, does PHECC [Regulator] say they are relevant?”
• “There should be a central hub”	
• “Should PHECC [the Regulator] not have these on their website?”	
**Suggestions:**	**Suggestions:**
• “Perhaps other voluntaries could all come together across the country and have CPC days.”	• “EMTs mentoring or assisting on EMT courses should get CPC points”
• “There should be regional training centres for CPC – organisational”	
• “Linking CPC points with your work, if you are a first aider in work and you have to treat someone in work should that count?”	
• “We are charged quite a lot for voluntary members to do CPC, could we be subsidised to help us with the costs?”	
• “Regional training days, you should utilise EMTs to instruct and teach and to gain CPC points”	
**Clarification:**	**Clarification:**
• “What if you are involved in community responder schemes and can those casualties be counted as your patient contacts because it doesn’t say it in the book?”	• “It says the student must document evidence by a paramedic or AP, the likes of EMT who are assisting EFR and CFR the likes of that you are not going to get a paramedic or advanced paramedic to say you assisted, or does that not count is that relevant will you get credit for training/helping?”
• “If we were to participate with paramedic class exercises, major incidents exercises *etc.*, could we get CPC points?”	• “And we start this from last November?”
• “Maybe different points for seminars, if you are only there for a couple of hours or a few days, what counts?”	• “So any training of any class counts, is that right?”
• “Seminar CPC points should not be standard, more clarification on points PHECC [Regulator] should allocate points for conferences *etc.*”	• “So you don’t have to put points down, just all the stuff we do?”
	• “The emergency CPC online where does that fall on-line? How can you prove how many hours you’ve done on it? What’s there to say you could log on and do more?”
	• “Is there a limit to how many points you can get for an activity?”
	• “You have to get 8 points from related activities is it compulsory that we can’t get all of them together is it compulsory that it’s a mix?”
	**Comment or unrelated question to the question:**
	• “Have the organisation considered how many more EMTs they will be training or CPC days?”
	• “The duty on its own is no use to you, if you don’t get patients”
**Question 4: Learning Portfolio: What is your understanding of what a learning portfolio is?**	
**Student EMTs**	**Experienced EMTs**
**Definition of Portfolio:**	**Definition of Portfolio:**
• “Put your certificates into it”	• “A record of activities”
• “Something to show evidence of what you’re doing to maintain competence”	• “Something to retain to show evidence of your yearly practice”
• “A folder which you keep to show evidence of practice”	• “A folder you keep to be able to reflect on and show what you’ve been doing”
• “A record of all that you do in the previous year”	
**Clarification required:**	**Benefits of a Portfolio:**
• “Who do you submit it to”	• “It helps you learn what you’ve done, you can reflect”
• “How long do you need to keep your portfolio for”	• “A good way of looking back and learning”
• “Where is the portfolio kept do you have to keep it or is it on line”	• “It’s on the 3 years that we do that is, over the 3 years”
• “In regard to confidentiality and data protection what must we do”	• “You can identify areas you need to improve on”
• “If you were managing a patient on the street could the ambulance sign you off for managing the patient before they arrived?”	
**Suggestions**	**Suggestions:**
• **:**“If you had a template for the portfolio”	• “What about CPC on-line learning portfolio?
• “PHECC [Regulator] should have a web page with all the CPC information”	
• “There should be a hub or webpage with all CPC information, conferences, templates, information”	
• “there could be a phone app that would give you notifications about CPC activities that are on”	
• “A Gmail calendar to notify you of events on your phone”	

## Results

Responses provided by the participants are detailed below, and are presented in the context of each of the individual questions posed, as per the coding framework used for data analysis. Selected responses from cohorts are presented in Table 1.

### Question 1: Is CPC a good idea?

The recent introduction of compulsory continuous professional competence for Irish emergency medical technicians was unanimously accepted as a positive initiative by these five groups who indicated this by a show of hands.

Most answers to this question related to the benefits of EMTs maintaining competence and both cohorts demonstrated through their responses that this was at the core of the initiative: “it keeps you up-to-date”; “it is absolutely necessary, it is a good thing”.

### Question 2: What problems do you foresee in completing CPC, and Question 3: what is unclear about the related activities section of the CPC booklet for EMTs?

(Supplementary information for Question 3 is presented in Table 2).

**Table 2:** Supplementary information for Question 3

**From the ‘Related Activities’ section of the CPC Guidance booklet for EMTs**		
**Activity**	**CPC points**	**Evidence**
CPC related training programme provided by training organisations or programmes accredited by other professional organisations (for example, An Bord Altranais (nursing regulator), Irish College of General Practitioners (ICGP) and so on)	1 point for each hour	Certificate
Case study	2 points	Case study on an incident, condition or injury encountered
Reflection on the incident	2 points	A document containing the main points learned
Seminars and conferences	1 point for each hour	Details of the seminar attended with a review of the key points learned
Programmes such as ACLS, PALS, PHTLS, PEPP, ATC, MIMMs, ITLS, Wilderness-EMT, ATLS, AMLS and so on	1 point for each hour	Certificate
Journal article review	2 points	Critical appraisal of a journal article
Electronic learning/on-line learning – related to practice	1 point for each hour	Printed certificate from site
Mentoring a student or being mentored on any experiential/operational ambulance, response vehicle placement.	1 point for each hour	Documented evidence of placement, signed by a paramedic or advanced paramedic

### The need for describing what constitutes ‘patient contact’

The CPC EMT guidance booklet outlines the requirement for EMTs to show evidence of at least 12 patient contacts and, therefore, the topic of ‘patient contacts’ was brought up by the groups. Indeed, in discussing the challenges associated with completing CPC, it was noted that this topic generated the greatest level of participant engagement and level of enthusiasm. It was clear from the comments made that participants were expressing frustration with respect to pragmatic difficulties that they had encountered in trying to fulfil their CPC requirements.

Arising from this discussion, it was evident that both the experienced and student cohorts were seeking clarification on a number of topics. These related to: duties; number of patient contacts; and definition of what constitutes a ‘patient contact’.

### Duties and patient contacts

The participants’ difficulties related primarily to their ability to experience and access frontline ambulance services and patient engagement; to experience diversity of patient types and scenarios; and to overcome limitations relating to the points above due to their geographic location (*e.g.*, urban versus rural). As one participant stated: “we have ten EMTs in our county, that’s 120 patients a year [we need to encounter], it just won’t happen”. To give further context, another EMT commented: “…we are getting plenty of duties, we are just not getting the patients, and so unless we start pushing people off horses or something, we are not going to get patients”. However, frontline ambulance placements alone were not seen to be the solution: “What if we go to events but don’t get any patient contacts?”, thus, highlighting the importance of not just accessing frontline ambulance experience but that such access must also be accompanied by the potential for more patient encounters.

Whilst the quantity of patient contacts required generated discussion and concern, so too did the type of patient contacts. In other words, what counts as a patient contact? One participant asked: “the evidence of at least 12 patient contacts per year is that just 12 plasters [band-aids/dressings] on 12 patients, what type of evidence do you need to show?” Another suggested that perhaps there should be “…more points for quality of care”.

One participant suggested that perhaps consideration should be given to allowing more CPC points for the more serious interventions performed or patient conditions encountered: “what about scoring higher points if you have more serious casualties, as well as points for skills?”

This sub-division of ‘patient contact’ gave rise to significant discussion and emphasised the link between the requirement for patient contacts and the need to increase the exposure of EMTs to other statutory front-line ambulance services provided by paramedics and advanced paramedics.

### The role and function of the regulator

Clarification regarding the role and function of the Regulator was identified as a further theme. Both groups suggested a more active role for the Regulator was necessary in co-ordinating CPC activities: defining ‘patient contact’; information focal point *i.e.* one website or webpage dedicated to providing information on CPC events/activities; awarding credit to CPC activities; and the ability to update registrants on their CPC requirements.

The administration of CPC by the Regulator was also challenged. In addition to the need for a central information hub for CPC, some participants believed that the Regulator should coordinate and even validate courses and CPC activities, as this is not the case currently: “where are the courses…. how would you check that they are relevant, does the Regulator say they are relevant?”; “more clarification on CPC points, the Regulator should allocate points for conferences *etc.*”; “do you see the Regulator organising ambulance placements for us?”. The booklet, according to one participant, “does not tell us where to go to complete CPC courses”.

EMTs are also required to provide evidence of case studies and reflective practice and so participants suggested that the Regulator should provide sample case studies; templates for case studies, reflective practice and learning portfolios. Participants agreed that templates should be provided: “on the case study [part of the requirements] there should be key points or a template provided, for it and for reflective practice”; “examples of what should be in your portfolio would be good”. Participants were keen for the Regulator to provide a clear outline of how their portfolios will be audited and by whom: “who signs off on our points?”; “how do you know if I haven’t made it up?”; “what is the mechanism for audit?”; “who will audit the whole process of CPC?”

### Question 4: Learning Portfolio: what is your understanding of what a learning portfolio is?

As part of their CPC requirements, EMTs must now maintain a Learning Portfolio: ‘The learning portfolio is a tool to support practitioners to commit and engage in lifelong learning…’[1]

With respect to the Learning Portfolio, participants were predominantly positive. No group objected to using or maintaining a portfolio and linked the learning portfolio to its intended aim: documenting experiences of required CPC activities.

However, there were some uncertainties regarding the administration of portfolios. Specifically, who will audit portfolios, to whom are they submitted, and the lack of a standardised sample template to be followed. Further questions relating to the portfolio included: “how long do you need to keep your portfolio for?”; “where is the portfolio kept, is it on-line?”

## Discussion

It was established that all five groups unequivocally support the introduction of CPC, however, there is a requirement to provide clarification in specific areas relating to the required CPC activities. From the fora, it can be determined that some of the most challenging or questionable areas relate to: the requirement to complete twelve patient encounters; what is classed as a patient encounter and how to achieve this. This, they suggest, could be facilitated if front-line ambulance placements were made available to them. However, ambulance placements by themselves may not be adequate unless the placements are in a busy area, in order to maximise the potential for varied and numerous patient contacts.

### Patient contacts and on-the–job learning facilitated through front-line ambulance placements

The facilitation of front-line ambulance placements could have many benefits, and not just related to more patient contacts. Placing EMTs with other professional colleagues would also provide the opportunity for additional experience and learning. There is value in such on-the-job learning, whilst there may be considerable difficulties in evaluating its effectiveness [25]. Regehr and Mylopoulos [26] suggest that education research emphasises the importance of context-based learning: *if we shift our perspective from a focus on education to a focus on learning, we will be able to direct additional efforts at understanding how professional learning not only arise from practice, but actually occurs in practice and is informed by practice.* These authors further suggest that apart from formal continuing educational delivery practitioners should learn from their own personal experiences of solving problems in their daily practice. Irish pre-hospital practitioners support the practical-based and operational context of learning [2, 17] and international evidence also emphasises the importance of work-based learning [27–31].

It would seem reasonable to suggest that EMTs are in favour of more patient contacts, not only to fulfil their CPC regulatory requirements but also to increase their interaction as pre-hospital practitioners and, thus, gain further experience. This is supported by their responses: “it is very important for EMTs to get ambulance placements”; “Do you see PHECC [Regulator] organising placements for us with HSE [Health Authority] so that we can get experience to build up our patient contact?” Their comments support the findings of previous studies, which demonstrated the preference of EMTs for participation in frontline ambulance duties with paramedics/advanced paramedics [2].

One of the major threats to patient safety in the pre-hospital environment is errors in decision-making and clinical judgement [32, 33]. Increasing their exposure to managing patients, through placements on frontline emergency ambulances or in an appropriately supervised relevant hospital department, would improve confidence and improve their ability in decision-making.

Linked to patient interaction is the ability to generate case studies and to encourage reflective practice as part of the CPC process. Therefore the more patient contacts the greater the opportunity to complete more case studies and encourage more reflective practice. This is supported by comments from our participants who questioned: “How do we get a case study, we need patient contacts and a case study may be difficult for us?”

### The learning portfolio

The learning portfolio is a collection of material that reflects on key events and processes in a professional’s career [34]. By ensuring a learning portfolio is an integral part of the CPC process, the practitioner can not only demonstrate evidence of proficiency but also should be able to identify areas for improvement or for future enhancement. Self evaluation is an essential part of continuous development [35]; the competent practitioner pursues lifelong learning through the recognition of deficiencies and the formulation of appropriate learning goals. Hence, the ability to assess one’s strengths and weaknesses is critical to the enterprise of lifelong learning [36]. There is a requirement for reflection and maintaining a learning portfolio. Reflection appears to be the ‘engine’ that shifts surface learning to deep learning [37] and transforms knowing in action into knowledge in action [38]. However, to encourage portfolio development, case study completion and reflective practice there is a need to address the current deficit of examples and information to maximise the proposed effect.

In our study, both cohorts appear to have a reasonable understanding of what a Learning Portfolio is and what it should be used for. However, again, they would like to see samples and clarification of what the Regulator would like to see them include in their learning portfolios.

### The role and function of the regulator – the Pre-Hospital Emergency Care Council (PHECC)

Participants believed that the Regulator could do more to assist with the effective implementation of CPC. This theme involved a substantial amount of interaction and response and detailed a considerable number of concerns. The areas of concern stated most frequently related to the Regulator not providing clarification on specific areas of CPC activities; not supplying templates for case studies or reflective practice; not providing portfolio requirements or samples; not providing a facility that directs registrants to national CPC programmes; and not providing details about auditing of CPC portfolios.

It would not be unusual for a Regulator of a healthcare profession or professions to provide information on Continuous Professional Competence/Education/Development. Many Regulatory bodies have a dedicated department to manage queries, provide the detail of audit and to interact generally with registrants. The Health and Care Professions Council (UK) (HCPC) have considerable resources related to Continuous Professional Development (CPD): information documents; templates; CPD video guides; audit process video; constructing your CPD profile video; glossary of terms; CPD evidence examples; CPD activity examples; audit dates; Standards of CPD; and a section on Frequently Asked Questions [5]. However, the HCPC is a multi-healthcare professions regulator with 16 professions and over 320,000 registrants and a much larger Regulator than the Irish Regulator. In Ireland, the Health and Social Care Professionals Council, has published a CPD Framework document as a template for each of their proposed registrant professional groups [39] but this does not include pre-hospital practitioners. Of note, guidance provided in their document contains information pertinent to CPD: templates; audit process; assessment outline; examples and supporting documentation. Furthermore, the Irish Medical Council, the body responsible for registering doctors, provides guidelines on the maintenance of professional competence and gives an outline of medical practitioners’ requirements to enrol in professional competence schemes accredited by the Medical Council [40].

Overall, there appears to be a consensus between both cohorts in our study that further clarification in the areas described above should be addressed by the Regulator (as done by the HCPC and Medical Council) to allow an effective model of CPC to be maintained and standardised amongst participating cohorts.

### Study limitations

As these were large fora comprising five groups with a total of 193 EMTs, this may have prevented everyone from participating in the discussions. Also, as the questions presented were pre-selected, these specific topics may have restricted other topics from further discussion. While the group was drawn from many regions, it was representative of only one EMT organisation, the Civil Defence, and while key areas were identified for further clarification or requiring additional information from the Regulator, the views are only that of one organisation’s members.

## Conclusion

This is the first study to consult with pre-hospital practitioners in Ireland, after the introduction of a CPC model. It demonstrates the similarities between what student EMTs perceived as practical challenges to the introduction of CPC, as they had not actively encountered CPC at that time due to their student status, and the actual practical challenges encountered by the experienced EMT groups. The study identified no evident difference between the stated views of either cohort and, in fact, may emphasise the need for the Regulator to consider reviewing the current iteration of CPC requirements for EMTs. While the introduction of Continuous Professional Competence requirements for EMTs in Ireland is a positive step by the Regulator, and is welcomed by the EMTs in this study, there remains some work to be done to refine the process. EMTs are keen to meet the requirements but need the Regulator to provide more information, examples and further clarification. It would appear that EMTs are motivated and accept CPC but further guidance from the Regulator could maintain and support such an initiative. Further research with professional groups could guide the appropriate revisions of this CPC model to be suitable for all pre-hospital practitioners, including those not currently required to maintain CPC such as paramedics and advanced paramedics.
